# Foxp1 Regulates the Proliferation of Hair Follicle Stem Cells in Response to Oxidative Stress during Hair Cycling

**DOI:** 10.1371/journal.pone.0131674

**Published:** 2015-07-14

**Authors:** Jianzhi Zhao, Hanjun Li, Rujiang Zhou, Gang Ma, Joseph D. Dekker, Haley O. Tucker, Zhengju Yao, Xizhi Guo

**Affiliations:** 1 Bio-X Institutes, Key Laboratory for the Genetics of Developmental and Neuropsychiatric Disorders (Ministry of Education), Shanghai Jiao Tong University, Shanghai, 200240, China; 2 Institute for Cellular and Molecular Biology, University of Texas at Austin, Austin, Texas, United States of America; National University of Ireland Galway, IRELAND

## Abstract

Hair follicle stem cells (HFSCs) in the bugle circularly generate outer root sheath (ORS) through linear proliferation within limited cycles during anagen phases. However, the mechanisms controlling the pace of HFSC proliferation remain unclear. Here we revealed that Foxp1, a transcriptional factor, was dynamically relocated from the nucleus to the cytoplasm of HFSCs in phase transitions from anagen to catagen, coupled with the rise of oxidative stress. Mass spectrum analyses revealed that the S468 phosphorylation of Foxp1 protein was responsive to oxidative stress and affected its nucleocytoplasmic translocation. *Foxp1* deficiency in hair follicles led to compromised ROS accrual and increased HFSC proliferation. And more, NAC treatment profoundly elongated the anagen duration and HFSC proliferation in *Foxp1*-deficient background. Molecularly, Foxp1 augmented ROS levels through suppression of Trx1-mediated reductive function, thereafter imposing the cell cycle arrest by modulating the activity of p19/p53 pathway. Our findings identify a novel role for Foxp1 in controlling HFSC proliferation with cellular dynamic location in response to oxidative stress during hair cycling.

## Introduction

Hair follicles undergo cyclic bouts of growth (anagen), regression (catagen) and rest (telogen) throughout life in mammalians [1,2,3]. Hair follicle stem cells (HFSCs) in the bulge niches are responsible for regeneration of various hair follicle cell lineages within well-defined anatomical compartments[4]. At the telogen phase, HFSCs are mostly maintained quiescently. At early anagen, HFSCs and their close progenies, hair germ (HG) cells, are activated by dermal papilla (DP), a cluster of underlying mesenchymal cells[5]. As hair growth progresses, HFSCs develop into the upper half of the slow-amplifying outer root sheath (ORS) and into the lower half of the fast-cycling ORS[6]. Meanwhile, matrix cells (Mx) at the base of the hair follicle divide rapidly but transiently prior to migrating upwards to form the hair sheath. Once having entered anagen V and catagen phases, HFSCs and their progenies cease cell proliferation and progressively return to quiescence[7].

The quiescence of HFSCs is precisely regulated by an integrated network consisting of extracellular (eg. Wnt, Bmp, Fgf, and Shh) signals from niches[5,8,9,10,11,12,13,14], as well as intrinsic programs governed by transcriptional factors, including NFATc1, Lhx2, Tcf3, and Sox9[15,16,17,18,19,20]. Once exiting from resting phase, HFSCs undergo proliferation with a limited number of cell cycles, and cease cell proliferation at late anagen and catagen[7]. At the end of catagen, the upper half of ORS cells return to quiescence and provide the major source of HFSCs in the new bulge[6]. In contrast to the accumulated knowledge of the network regulating HFSCs quiescence, it is unclear for the mechanism controlling the pace and capacity of HFSCs proliferation.

Reactive oxygen species (ROS) are usually formed as byproducts of the normal metabolism of oxygen in aerobic cells. ROS was originally thought to be toxic, leading to DNA damage, protein oxidation and lipid peroxidation. However, ROS was recently shown to support stem cell self-renewal during spermatogenesis[21]. ROS also has been demonstrated to be a second messenger for a variety of signaling pathways that regulate cell proliferation, differentiation and senescence[22,23]. In addition, ROS modulates transcriptional programs of Foxo and p53 proteins and imposes cell cycle arrest or apoptosis[24,25]. Since the epidermis is the outer barrier of the body and is frequently exposed to UV-induced oxidative stress, ROS signaling leads to various deleterious effects, including melanocyte apoptosis in hair graying [26] and skin cancer[27,28,29]. Yet, little is known regarding a potential involvement of ROS in hair cycling and HFSCs proliferation.

Foxp1 regulates multiple cell differentiation processes[30,31,32,33,34,35] and is required to maintain quiescence of HFSCs via regulation of *Fgf18*[36]. In this study, we identified a progressive rise of ROS levels in HFSCs at phases from anagen to catagen, coupled with a nucleocytoplasmic shuttling of the Foxp1 protein. We have revisited the epidermis-specific *Fox*p*1*-deficient conditional mice and have detected impaired ROS accrual in hair follicles accompanied by an increase of HFSC proliferation. Therefore, our observations also provide the novel role for Foxp1-tuned ROS signaling adopted by HFSCs for the control of their proliferation during anagen phase.

## Materials and Methods

### Mice

To generate the *Foxp1* conditional knockout mice, we mated *K14-Cre* [36] and *Foxp1* ^*fl/fl*^ mice[31]. The heterozygous *K14-Cre; Foxp1* ^*fl/+*^ progeny were bred subsequently with the *Foxp1* ^*fl/fl*^ mice to get homozygous *cKO* mutant. The male *K14-Cre; Foxp1* ^*fl/fl*^ mice are sterile, therefore male *Foxp1* ^*fl/fl*^ mice were bred with the female *K14-Cre; Foxp1* ^*fl/+*^ mice. Haircut was carried out on anaesthetized mice by electric scissors.

Mice were bred in specific pathogen-free environment and caged in groups less than eight. During housing, animals were cleaned twice a week. HE staining, IHC detection and qRT-PCR and western blot analyses were each conducted using 3 pairs of animals. For FACS analyses, 4–6 pairs of animals were used. All animal procedures in this study were performed in accordance with recommendations in the National Research Council Guide for Care and Use of Laboratory Animals, with the protocols approved by the Institutional Animal Care and Use Committee of Shanghai, China [SYXK (SH) 2011–0112]. All efforts were made to minimize suffering and mice were euthanized by carbon dioxide in a closed cage. The mice were sacrificed at specific times for different experiments which were annotated in the text. A completed ARRIVE guidelines checklist is included in S1 Checklist.

### Cell and cell culture

The HaCat line is the keratinocyte cell from human skin and purchased from the American Type Culture Collection (ATCC, Rockville, MD). In our research, we used this cell line to study the subcellular location of Foxp1 protein. Due to the low transfection efficiency of HaCat cells, we used HEK293T cells to perform western blot. The CHO cell line is the Chinese hamster ovary cells. In order to examine the modification of mouse Foxp1 protein under oxidative stress, we choose a mouse CHO cell line instead of human 293T cells. The HeLa cells that were tolerant to oxidative stress were used here is to test the role of Foxp1 in antagonizing Trx1 function. Besides, cells were cultured in high DMEM medium added with 10% FBS, 1% penicillin, and 1% streptomycin at 37°C and 5% CO_2_.

### Plasmids, qRT-PCR and western blot

Foxp1-His was constructed on vector pcDNA3.0. Foxp1-EGFP, Foxp1-NLSm-EGFP, Foxp1(S468A)-EGFP and Trx1-RFP were cloned into the pCMV-TNT vector. Truncated or site-directed mutation of Foxp1 was generated with PCR by specific primer design. pcDNA3.0-Foxp1-His and pcDNA3.0-Trx1 were used in the co-IP experiment and ROS detection *in vitro*.

Total RNA from skin epidermis was isolated using TRIzol Reagent (Invitrogen, 15596–026, USA). cDNA was then synthesized using the Reverse Transcription System (Promega, A3500, USA). The quantitative RT-PCRs were performed on an Applied Biosystems 7500 using SYBR reagents (Roche, 04913850001, Switzerland). Primer sequences are listed in Supporting Information.

Protein samples from skin epidermis were subjected to western blot analysis using standard procedures. The primary antibodies used were: anti-Foxp1 (Millipore, ABE68, USA, 1:1000); anti-phospho serine/threonine/tyrosine (Abcam, ab15556, UK, 1:1000); anti-p53 (Cell Signaling, 2524, USA, 1:1000); anti-p-p53 (Cell Signaling, 9284, USA, 1:1000); anti-β-actin (Santa Cruz, sc-81178, USA, 1:1000); anti-p19^ARF^ (Santa Cruz, sc-32748, USA, 1:1000). Secondary antibodies coupled to HRP used were: HRP-Goat anti-Rabbit (Santa Cruz, sc-2030, USA, 1:5000); HRP-Goat anti-Mouse (Santa Cruz, sc-2005, USA, 1:8000) and HRP-Goat anti-Rat (Santa Cruz, sc-74088, USA, 1:5000).

By the way, skin epidermis samples analyzed here were collected from mice that were euthanized prior to isolation.

### HE staining and IHC analyses

For paraffin sections, back skin tissues picked from the identical site were fixed in 4% paraformaldehyde (PFA) overnight at 4°C and embedded in paraffin. For HE staining, paraffin sections were dewaxed, rehydrated and stained with hematoxylin and eosin. For immunohistochemistry (IHC), sections were usually subjected to antigen unmasking in 10 mM Citrate (pH 6.0). For DAB staining, H_2_O_2_ was necessary to block peroxidase. The primary antibodies applied were: anti-CD34 (Biolegend, MEC14.7, USA, 1:100); anti-Foxp1 (self-made, Abmart, FR, 1:100) (S1 Fig); anti-BrdU (CST, 52925, USA, 1:100); anti-Trx1 (Santa Cruz, sc-20146, USA, 1:50). Secondary antibodies used were: Fluor 594 Goat Anti-Rat IgG (H+L) (Invitrogen, A-11007, USA, 1:200), Fluor 488 Goat Anti-Mouse IgG (Invitrogen, A-31561, USA, 1:200), Fluor 594 Donkey Anti-Rabbit IgG (H+L) (Invitrogen, A-21207, USA, 1:200) and Fluor 488 Donkey Anti-Rabbit IgG (H+L) (Invitrogen, A-21206, USA, 1:200).

### BrdU pulse-chase, mass spectrum analyses

For BrdU (Sigma-Aldrich, B5002, USA) pulse-chase experiments, mice were injected intraperitoneally with 50 μg/g once or twice a day and then chased after a period of time. For paraffin sections, treatment with 1 N HCl for 20 minutes and then neutralization with 0.1 M sodium borate (PH 8.5) for 10 minutes are necessary before proceeding with IHC analysis by anti-BrdU (CST, 52925, USA, 1:100). For GC-MS, Foxp1 protein was over-expressed in CHO and 48 hours later H_2_O_2_ stimulus was applied for two hours. The cells were then lysed in NP40 lysis buffer with additional protease inhibitor cocktail (Sigma-Aldrich, P8340#, USA). SDS-PAGE separation and silver staining were used to get the gel with single Foxp1 protein. Through destaining, reduction, enzymolysis and ultrasonication, the protein sample was then dissolved in solution A (98% ddH_2_O+2% CH3CN+0.1% Formic Acid) and analyzed by Gas Chromatograph-Mass Spectrometry (GC-MS) (Agilent, 7890A-5975C, USA).

### FACS analyses

The protocol and antibodies used here are referred to in the work by Elaine Fuchs’ lab [37]. After removing the subcutaneous fat and blood vessels from the skin with a scalpel, we floated the whole skin with an unsubmerged epidermis on trypsin solution (Gibco, 27250–018, USA) overnight at 4°C. The next day, we collected the epidermis by gently scraping it away from the dermis and repeatedly pipetted it until it was triturated. Single-cell suspensions were obtained through filtering with 70 μm and 40 μm strainers consecutively. Cell suspensions were then incubated with the appropriate antibodies for 30 min at 4°C and shaken gently every 10 min to avoid precipitation. FACS was performed on the BD Calibur flow cytometer and FACS analyses were processed with the FlowJo program. Antibodies used were: anti-CD34–Biotin (eBioscience, RAM34, USA), anti-a6-integrin–percp efluo710 (eBioscience, eBioGoH3(GoH3), USA). For ROS evaluation, we incubated the single-cell suspensions with CM-H2DCFDA (Invitrogen, 6827, USA) in a 5 μM final concentration at 37°C for 45 min and directly proceeded to cell analysis without washing.

### Statistics

Statistical analysis was performed by Student’s t test using GraphPad Prism 5 software. Data are represented as means ± SEM and significance was set at p ≤ 0.05.

## Results

### Foxp1 is relocated from nucleus to cytoplasm in HFSCs from anagen to catagen accompanying the rise of ROS levels

**Fig 1:**
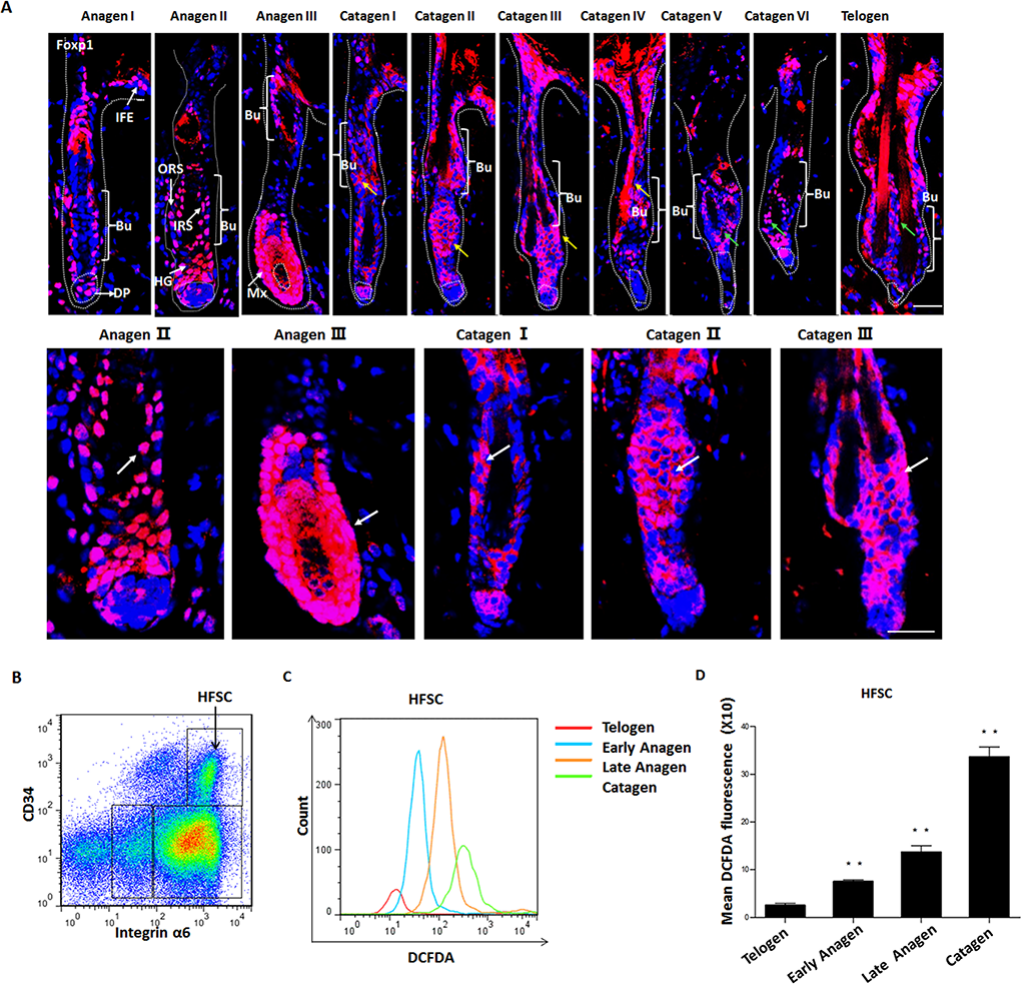
Foxp1 is translocated from the nucleus to cytoplasm of HFSCs at phases from anagen to catagen accompanying with rise of ROS levels. **A:** Foxp1 distribution among distinct populations of hair follicles. Foxp1 was localized within nuclei from anagen I to III, exported to cytoplasm from catagen I to catagen V, and progressively relocalized into the nuclei at catagen VI and telogen. Lower panel was the high power view of upper panel. Scale bars: 50 μm. Blue, DAPI; red, anti-Foxp1. Abbreviations: Ep, epidermis; IFE, interfollicular epidermis; Bu, bulge; HG, hair germ; DP, dermal papillae; ORS, outer root sheath; IRS, inner root sheath; HF: hair shaft. **B:** Representative dot plot of FACS for HFSCs identified by CD34^+^/Integrin α6^+^ at early telogen (P49). Abbreviations: HFSCs, hair follicle stem cells. **C:** Histograms of DCFDA fluorescence intensities of HFSCs at telogen (P20), early anagen (P24), late anagen (P27) and catagen (P40) (n = 6, 6, 7, and 6, respectively). **D:** Quantification of (B) indicates a progressive increase in ROS levels in HFSCs from telogen to catagen. *, p<0.05; **, p<0.01.

Foxp1 has been reported to be widely expressed in varied hair follicle cell populations and HFSC[36]. To explore its function in other phases of hair cycling besides telogen, we first performed a thorough IHC examination for Foxp1 in hair follicles. The hair follicles at telogen, anagen and catagen were isolated from dorsal skin of mice at P21, P24-P27 and P40 respectively. The segmentation of each phase of hair follicle was assessed by morphological characteristics of each phase. The specificity of our self-made Foxp1 antibody was tested by IgG control in S1A Fig. The expression of Foxp1 in HFSCs was also evidenced by co-localization with NFATc1 (S1B Fig). During anagen I-III, Foxp1 primarily localized to the nuclei of a variety of cell populations, including bulge stem cells (Bu), ORS, the inner root sheath (IRS), HG, IFE and matrix (Mx) (white arrows in Fig 1A). However, Foxp1 progressively accumulated within the cytoplasm from catagen I to catagen V (yellow arrows in upper panel and white arrows in lower panel of Fig 1A). Then from catagen VI to telogen, Foxp1 localization was again enriched within the nuclei of bulge stem cells (green arrows in Fig 1A). These observations indicate that Foxp1 is relocated from nucleus to cytoplasm in HFSCs from anagen to catagen. Interestingly, we also detected a progressive rise of oxidative stress in HFSCs from anagen to catagen. The HFSCs could be characterized by FACS staining with CD34 and Integrin α6[37]. As showed by the dot plot, HFSCs were distinguished by CD34^high^α6^high^ (Fig 1B). Oxidative stress were evaluated by H2-DCFDA staining in combination with the CD34^high^α6^high^ markers. We observed a 2-3-fold increase, as determined by mean DCFDA fluorescence intensities in HFSCs during each phasic transition (Fig 1C and 1D).

### Dynamic relocalization of Foxp1 in response to oxidative stress

**Fig 2:**
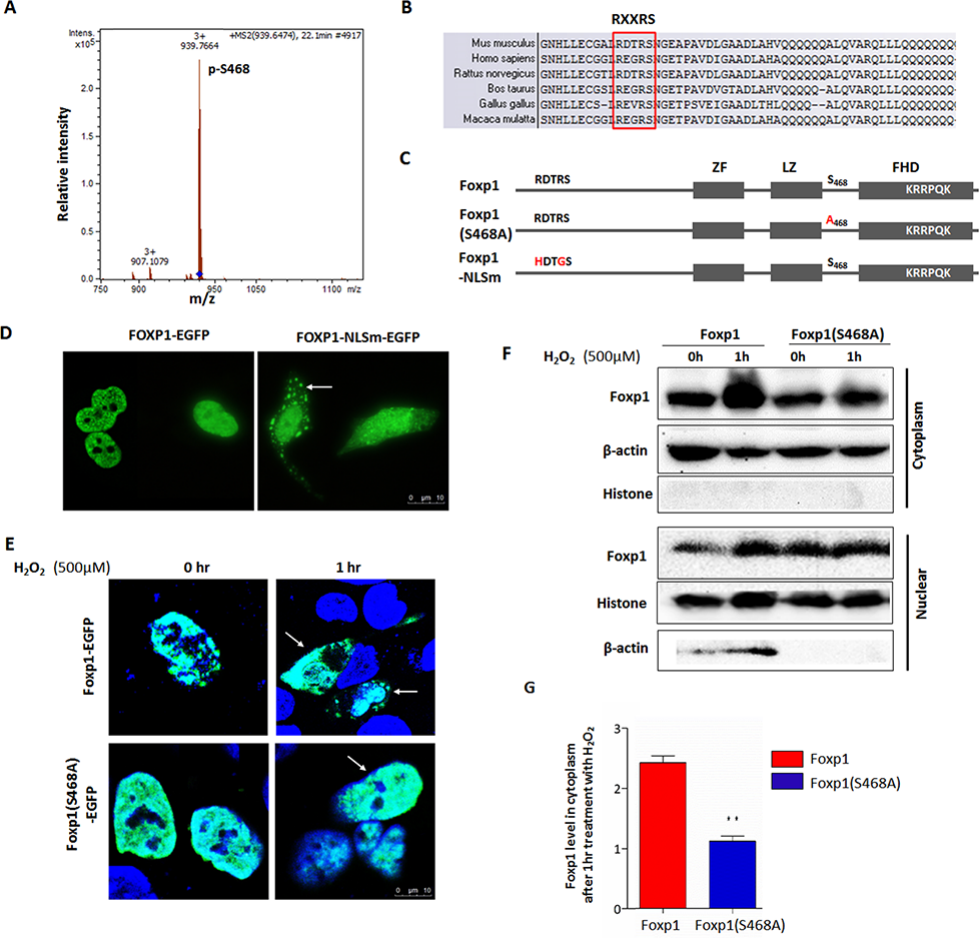
The dynamic nucleocytoplasmic localization of Foxp1 in response to oxidative stress. **A:** Identification by mass spectrum analysis of phosphorylation at Foxp1 S468 sensitive to 2-hour H_2_O_2_ stimulation in CHO cells. **B:** Alignment of Foxp1 N-terminal residues across multiple species revealed a conserved nuclear localization motif of RXXRS (boxed). **C:** Linear schematics of Foxp1 depicting mutations (in blue) of the RXXRS motif and S468. Zinc-finger (ZF), leucine-zipper (LZ) and forkhead domains (FHD) are indicated by boxes. In Foxp1N, RDTR is mutated to HDTG, leading to loss of function of NLS; in Foxp1(S468A), A is substituted for S, leading to loss of phosphorylation at S468. **D:** Representative images showing defective nuclear localization of the Foxp1NLSm-EGFP fusion protein (green) following transient transfection into HaCat cells. **E:** Representative images showing defective nuclear export of the Foxp1(S468A)-EGFP fusion protein in transfected HaCat cells following one-hour stimulation with 500 μM H_2_O_2_. Green, EGFP fluorescence; DAPI, blue; scale bar: 10 μm. **F:** 293T cells were transfected with Foxp1 or Foxp1(S468A) expression constructs. Western blot was conducted to evaluate the relative level of Foxp1 protein in cytoplasm or nucleus following one-hour stimulation with 500 μM H_2_O_2_. **G:** Quantification of the relative Foxp1 levels by gray scale in (F, n = 3).

Given the concurrence of the rise of oxidative stress and the nucleocytoplasmic shuttling of Foxp1 in hair follicles at phasic transition from anagen to catagen, we reasoned that might reflect a structural modification mediated by oxidative stress. When CHO cells were transfected with a Foxp1-expression construct and stimulated with 500 μM H_2_O_2_ for two hours, we detected a phosphorylation site at S468 in response to H_2_O_2_ stimulation by GC-MS analysis (Fig 2A). A putative nuclear localization motif (NLS; RE/DXRS), confirmed for the close paralogue Foxp3[38], is conserved in Foxp1 among various species (Fig 2B). To further address the mechanism by which Foxp1 undergoes ROS-mediated changes in subcellular localization, we mutated the canonical basic residues of the putative NLS (RDXRS/HDTGS), as well as the S468A amino acid in Foxp1 protein (Fig 2C).

Foxp NLS mutant fragment (Foxp1-NLSm) was fused with EGFP and transfected into HaCat cells. Fluorescence images indicated that the majority of Foxp1-NLSm-EGFP was retained within the cytoplasm, whereas wild type Foxp1-EGFP localized primarily to nuclei (arrow in Fig 2D). In addition, the Foxp1 mutant (S468A-EGFP) primarily localized within transfected nuclei of H_2_O_2_-treated HaCat human keratinocyte cells, whereas control Foxp1-EGFP distributed in both nucleus and cytoplasm (arrows in Fig 2E). In addition, the wild type Foxp1 and mutant Foxp1 (S468A)-expressing vectors were transfected in 293T cells and followed by one hour H_2_O_2_ stimulation. Western blot validated the relative increase of mutant Foxp1 levels in cytoplasm as compared to the wild type Foxp1-expressing construct (Fig 2F and 2G). These findings indicated that the S468A point mutation significantly impaired transport from the nucleus to the cytoplasm that was observed in response to high oxidative stress. Our identification of at least two key amino acid motifs regulating Foxp1 nuclear-cytoplasmic localization suggests that an intrinsic mechanism governs Foxp1 dynamic relocation in response to oxidative stress during hair cycling.

### Depletion of Foxp1 augments the fraction of HFSCs at S phase during anagen stage

**Fig 3:**
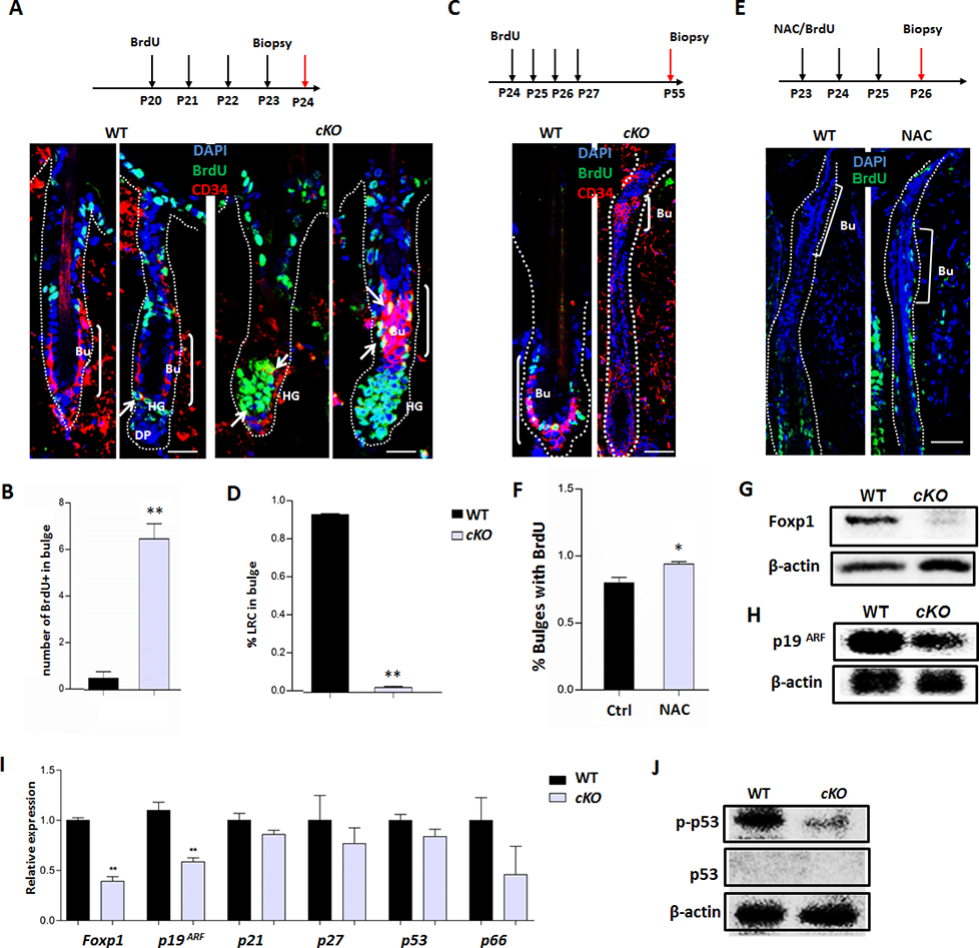
*Foxp1* deficiency augments the proportion of S-phase HFSC at anagen phase. **A:** IHC with anti-CD34 (red) and anti-BrdU (green) staining of hair follicles following 4-day BrdU pulse-chase in the *Foxp1* ^*fl/fl*^ (WT) and *K14-Cre; Foxp1* ^*fl/fl*^ (*cKO*) mice (P20-P23). The upper panel showed the timing of BrdU injection and sectioning. Abbreviations: Bu, bulge; HG, hair germ; DP, dermal papillae. Scale bars: 25 μm. **B:** Quantification of the number of BrdU^+^ cells in the bulges of (A). The *cKO* hair follicles at P24 displayed extensive BrdU^+^ cells in the hair germ and bulge cells, whereas the WT controls had few BrdU^+^ cells in the identical regions (n = 3,4). *, p<0.05. **C:** IHC for hair follicles at P55 following 28-day BrdU pulse-chase. The upper panel showed the timing of BrdU injection and sectioning. Scale bars: 75 μm. **D:** Quantification of the percentages of LRC in the bulges of (C). Few label-retaining cells (LRC) were detected in the bulges of *Foxp1 cKO* mice. n = 4; *, p<0.05. **E**: NAC treatment and BrdU injection once a day from P23 to P26 enhanced cell proliferation of HFSCs in WT early anagen. Scale bar, 50μm. **F:** Quantification of the frequency of BrdU^+^ cells in HFSCs in (E). *, p<0.05. **G-H:** Western blotting demonstrated a decrease of Foxp1 (G) and p19^ARF^ (H) protein levels within *cKO* hair follicles at anagen (P23). **I:** Down-regulation of *p19* ^*ARF*^ transcripts within *cKO* anagen (P23) hair follicles relative to the WT as determined by qRT-PCR. **J:** S15 phosphorylated-p53 protein level was relatively decreased within *cKO* anagen (P23) hair follicles.

Direct involvement of Foxp1 in the periodical ROS fluctuation during hair cycling was next evaluated using *K14-Cre; Foxp1* ^*fl/fl*^ (hereafter designated as *cKO*) mutant mice with conditional *Foxp1*-deficiency in the skin basal layer and hair follicles. Consistent with a previous report [36], loss of *Foxp1* led to shortened telegon duration and premature hair cycling. IHC examinations validated the loss of Foxp1 in hair follicles (S2 Fig). During the first postnatal hair cycle, obvious shedding was detected in the *cKO* mice at P29 (S3A Fig). The advanced hair cycling was more evident in the second cycle. At P45, the hair follicles in the wild type and the *cKO* mutant were both at telogen phase. By P55, the wild type hair follicles were still retained at telogen, whereas the *cKO* mice had already entered into the next anagen phase (Fig 3C and 3D). In addition, statistical analyses confirmed that the *cKO* mice also displayed shortened anagen duration (S3E Fig), as also evidenced by shortened hair shaft length at P47 (S3B Fig).

To investigate the cell cycle status in HFSC at loss of *Foxp1*, our *cKO* mice were pulsed with BrdU once a day from P20 to P23 and chased at P24. IHC with anti-CD34 and anti-BrdU was then performed to gauge cell proliferation rates of HFSCs (Fig 3A). As expected, the number of BrdU^+^ cells in the bulge and hair germ was significantly increased in *Foxp1 cKO* mice as compared to controls (Fig 3B). In consistent, when BrdU was pulsed between P23 to P27 and chased at P55 (ie, after one hair cycle), IHC analysis detected far fewer BrdU label-retaining cells (LRCs) in the bulges of *Foxp1 cKO* mice, whereas a number of the LRCs were identified in the bulges of wild type controls (Fig 3C and 3D). Intriguingly, when NAC was injected together with BrdU pulse-chase from P23 to P26, cell division rates were modestly increased as compared to controls (Fig 3E and 3F). This suggested that an increase of oxidative stress decreased the proportion of S-phase-HFSCs at anagen, then negatively regulated the HFSC proliferation.

To address the mechanisms underlying augmented HFSC proliferation, we examined the levels of several cell cycle regulators. Western blotting indicated that the expression of p19^ARF^ was markedly down-regulated at the anagen phase of *cKO* hair follicles, coupled with the loss of Foxp1 expression (Fig 3G and 3H). The decrease in *p19* ^*ARF*^ expression was validated by qRT-PCR, whereas the expression of *p21*, *p27*, *p53* and *p66* were relatively unaltered (Fig 3I, S1 Table.). We also observed decreased S15 phosphorylation of p53 in the *cKO* hair follicles, whereas no accumulation of pan-p53 was observed in either WT or *cKO* (Fig 3J). p53 is stabilized in HFs only in response to DNA damage-induced cell death[39]. In light of the well-established p19^ARF^/p53 axis regulating cell cycle arrest[40,41,42], the decrease of p19^ARF^ expression and S15 phosphorylation of p53 indicates that *Foxp1* loss relieves cell cycle arrest in anagen HFs, at least partially accounting for the effect of Foxp1 in promoting HFSC proliferation.

### Foxp1 deficiency impairs the accrual of oxidative stress during hair cycling

**Fig 4:**
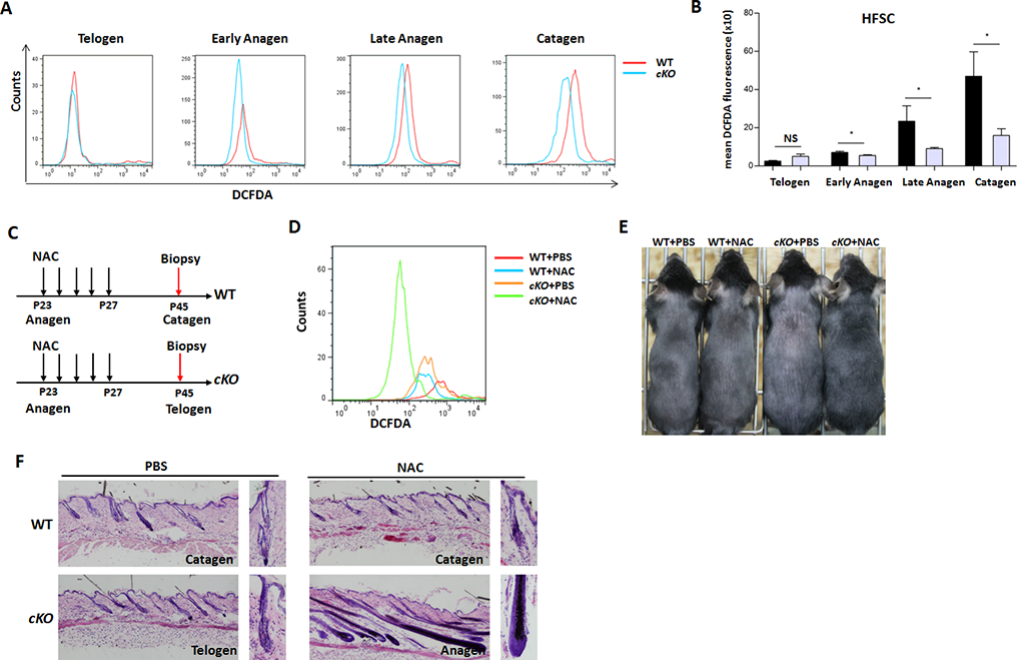
Deficiency of *Foxp1* in hair follicles impairs ROS accrual at anagen and catagen. **A:** Representative histograms of DCFDA mean fluorescence intensities (MFI) in HFSCs from *Foxp1* ^*fl/fl*^ (WT) and *K14-Cre; Foxp1* ^*fl/fl*^ (*cKO*) mice at telogen (P20), early anagen (P24), late anagen (P37) and catagen stages (P40) (n, 6–8). **B:** Quantification of the MFIs of (A) reveal decreased ROS levels in anagen and catagen in *cKO* mice compared to controls. NS, no significance; *, p<0.05. **C:** Flowchart depicting NAC treatment from P23 to P27 and sectioning for dorsal P45 HFs in mice. **D:** NAC treatment decreased ROS levels in HFSCs from both the WT and *Foxp1 cKO* mutant mice as determined by DCFDA staining/flow cytometry. **E:** Comparative dorsal views of the back hair follicles at P45 of cKO and WT mice as in (C). **F:** HE histological analysis for hair follicles at P45 as depicted in the flowchart of (C, n = 3–4).

To investigate the role of Foxp1 in ROS accrual at anagen and catagen phases, flow cytometry/H2-DCFDA staining was performed. Representative histograms of ROS levels in hair follicles were showed in Fig 4A. The *cKO* mice had statistically significant reduction of ROS levels in the HFSCs at anagen and catagen phases (Fig 4B). Only the ROS levels in the telogen HFs were not significantly decreased in the *Foxp1-*deficient mice as compared to the controls. These findings identify Foxp1 promotes the accrual of oxidative stress in hair follicles at anagen and catagen phases.

To investigate the correlation of Foxp1 and oxidative stress during hair cycling, anti-oxidant NAC was smeared on the backs of mice once a day at late anagen from P36 to P40, and the oxidative stress in the HFSCs of *cKO* mutant mice were both decreased by NAC treatment (Fig 4D *)*. Then NAC was injected once a day from P23 to P27 during early anagen, and the phases of hair follicles were examined at P45 (Fig 4C). In the *Foxp1 cKO* mutants, the reduction of ROS levels by NAC treatment arrested the hair follicles at the first anagen till P45—at time at which PBS-treated HFs had already entered telogen (Fig 4E and 4F). The additive effect of NAC and *Foxp1* deficiency suggests that the pace of HFSC proliferation is sensitive to oxidative stress.

### Foxp1 tunes oxidative stress through its interaction with Trx1

**Fig 5:**
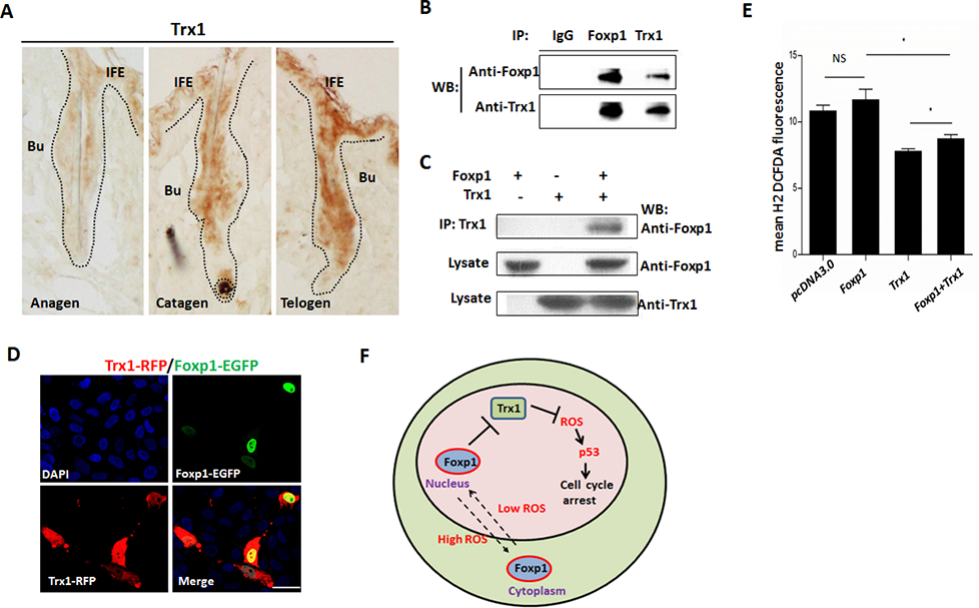
Foxp1 tunes ROS level through protein interaction with Trx-1. **A:** Anti-Trx1 IHC confirming extensive expression of Trx1 in HFs at anagen (P23), catagen (P40) and telogen (P55). **B:** Interaction of Foxp1 and Trx-1 endogenous proteins in anagen HFs as determined by Co-IP of protein lysates. **C:** Interaction of Foxp1 and Trx-1 following ectopic expression of Foxp1-His and Trx1 in transfected HeLa cells. Cell lysates were immunoprecipitated by anti-Trx1 antibody and detected by anti-His antibody. **D:** Colocalization of Trx1-RFP and Foxp1-EGFP protein within the nuclei (blue, DAPI) of HaCat cells. **E:** Flow cytometry of DCFDA-stained HEK293T cells following transient transfection of the indicated constructs (2 μg Foxp1 and/or 2 μg Trx1 expressing vector) indicated that Foxp1 releases inhibition of Trx-1-mediated ROS accrual. **F:** Model for the mechanism by which Foxp1 regulates redox homeostasis during hair cycling. Foxp1 is located within nuclei under conditions of low oxidative stress. Foxp1 suppresses the function of the Trx1 protein in decreasing ROS levels, and then imposes cell cycle arrest through p19/p53 axis. Foxp1 is exported into the cytoplasm when the ROS levels approach a high threshold.

Thioredoxin-1 (Trx1) acts as a reductive cofactor and is an important ROS scavenger in controlling gene expression of a number of redox-sensitive transcription factors, including NF-κB and AP-1[43]. IHC analysis revealed that Trx1 was extensively expressed in hair follicle cells in a pattern similar to Foxp1 (Fig 5A). Foxp1 could be immunoprecipitated by anti-Trx1 in hair follicles (Fig 5B), suggesting an additional protein-protein interaction of Foxp1 and Trx1. This was validated *in vitro* by Co-IP of the two endogenous proteins in HeLa cells (Fig 5C). In addition, HaCat cells were co-transfected with Trx1-RFP and Foxp1-EGFP fusion vectors. Although Trx1-RFP was extensively detected in both nucleus and cytoplasm, Foxp1-EGFP and Trx1-RFP still could be co-localized within nuclei (Fig 5D). These data indicate that Foxp1 functions as a binding partner with Trx1 protein in regulating redox hemostasis. Finally, we investigated the impact of Foxp1 on the anti-oxidative action of Trx1 under oxidative stress. As expected, HEK293T cells transfected with Trx1 displayed a marked decrease in ROS level under H_2_O_2_ stimuli; however, co-transfection with Foxp1 significantly released the inhibition of Trx1 on ROS accrual (Fig 5E). We conclude that Foxp1 regulates oxidative stress through antagonizing the reductive function of Trx1 by forming a protein complex.

## Discussion

Foxp1 has been reported to regulate HFSCs quiescence at telogen stage[36]. In this study, we uncovered a novel role for the Foxp1 protein in tuning the HFSC proliferation through augmenting oxidative stress during hair cycling. Our observations support the following conclusions: 1) Oxidative stress is progressively elevated in HFSCs during telogen to catagen transitions. 2) In response to oxidative stress, Foxp1 undergoes dynamic changes in nucleocytoplasmic localization with modification of S468 phosphorylation. 3) Loss of *Foxp1* augments HFSC proliferation and compromises oxidative stress. 4) NAC treatment profoundly elongates the anagen duration in *Foxp1*-deficient mice. 5) Foxp1 directly represses reductive function of Trx1 in regulating redox homeostasis through forming a complex. Collectively, these results provide a model in which sensitivity of Foxp1 to oxidative stress affects its relocation between the nucleus and cytoplasm of HFSCs. Conversely, nuclear Foxp1 augments oxidative stress via interaction with and functional suppression of Trx1, and then exerts cell cycle arrest through p19/p53 pathway during anagen of HFSCs (Fig 5F).

### Reciprocal interaction between Foxp1 and oxidative stress in HFSCs

Recently, one report presents that exclusive cytoplasmic localization of Foxp1 in endometrial adenocarcinoma is linked with deep myometrial invasion mediated by HIF-1α, which is an essential player in cellular and systemic responses to hypoxia[44]. It indicates a translocation of Foxp1 protein in response to oxidative stress. In this study, we identified the dynamical Foxp1 subcellular location in hair follicles. We detected a progressive increase of oxidative stress in HFSCs during transitions from telogen to catagen, which was coupled with the translocation of Foxp1 from nucleus to cytoplasm. The responsiveness of Foxp1 to oxidative stress was confirmed *in vitro* by H_2_O_2_ stimulation (Fig 3E–3G). And more, the hair follicle cells at catagen phase exhibited excessive oxidative stress and underwent extensive cell death, accompanying with a translocation of Foxp1 protein. HaCat cells treated by H_2_O_2_ also displayed extensive cell death characteristics, as evidenced by TUNEL staining (S4 Fig). Therefore, we could not exclude the possibility that the shuttling of Foxp1 from nucleus to cytoplasm is a consequence of DNA damage and cell apoptosis. MS analysis revealed that the dynamic nucleocytoplasmic shuttling of Foxp1 in response to oxidative stress is regulated, at least in part, by specific phosphorylation at S468. We also identified the “RXXRS” domain as a bona fide NLS that is conserved in Foxp3 and critical for its nuclear entry[38]. Therefore, some common mechanisms might be employed by Foxp1 and Foxp3 in regulating their cellular localization in response to external stimuli.

Another key finding is that Foxp1 has a novel role in buildup of oxidative during the growth phases of hair cycling. Loss of *Foxp1* in the epidermis led to impaired oxidative stress accrual in anagen and augmented proliferation of HFSCs. Therefore, Foxp1 acts as a pro-oxidant to tune-up oxidative stress in regulating hair cycling. Of course, we still could not exclude the contribution of *Foxp1-Fgf18* pathway in regulating HFSCs proliferation during anagen phase[6,11]. Foxp1 appears to regulate redox homeostasis through by antagonizing the function of Trx1. We propose that Foxp1 augments oxidative stress by suppressing the reductive function of Trx1 via protein-protein interaction.

### The negative effect of ROS signaling on HFSC proliferation during anagen

HFSCs are profoundly resistant to DNA damage-induced apoptosis[39]. However, the function of ROS signaling in regulating HFSC proliferation does not receive much attention in the past researches. We observed a progressive rise of oxidative stress in HFSCs during phasic transitions from telogen to anagen, which may be due to the higher mitochondrial content, higher metabolism in proliferating HFSCs at anagen phases relative to telogen phases. Our observations indicated that *Foxp1*-deficiency or NAC treatment modestly increased the HFSC proliferation at anagen (Fig 3E and 3F). The NAC treatment in *Foxp1*-deficient mice profoundly elongated the anagen duration and HFSC proliferation. Therefore we speculate that oxidative stress also influence HFSC proliferation as a negative regulator. Molecularly, impairment of the p19^ARF^/p53 cascade, which regulates cell cycle arrest [40,41,42], is likely one of the molecular pathways mediating the influence of ROS signaling on HFSC proliferation. Loss of *Foxp1* in hair follicles at anagen also led to a decrease in expression of p19^ARF^ and in phosphorylation of p53 at S15. Given that the TRR-Trx1 system controls p53 stability and activity[45], the effect of Foxp1 on p53 activation may be mediated indirectly through its interaction with Trx1.

The ROS signaling has been reported to regulate stem cell quiescence and proliferation in a variety of stem cell populations with stage- and dose-dependent manner. For instance, a hypoxic niche environment with low oxidative stress better maintains HSCs self-renewal[46], whereas moderate oxidative stress sustains HSC proliferation, differentiation and mobilization[47,48]. Nevertheless, high oxidative stress is toxic for HSCs and result in senescence[49][50]. In contrast, high oxidative stress is required for self-renewal of adult stem cells including neural and spermatogonial[51,52]. Taken together with our findings, it is conceivable that ROS signaling maybe physiologically link to additional pathways governing hair growth and regeneration, which deserves to be further clarified.

In summary, we have dissected a hitherto undescribed and essential function for the Foxp1 protein in regulating HFSC proliferation through tuning ROS signaling during hair cycling. Gain, loss, or translocation of *Foxp1* is associated with multiple cases of carcinogenesis[33,34,53,54,55]. Given that one of the most important underlying oncogenic mechanisms is the accumulation of ROS-induced DNA damage and subsequent mutagenesis, our findings may provide novel cues for understanding the potential interaction of Foxp1 and ROS signaling in multiple malignancies.

## Supporting Information

S1 Checklist A completed ARRIVE guidelines checklist of animal research. The research on animal ethical statement was did through each paragraph of the manuscript.
(DOCX)

S1 Fig Foxp1 is located in HFSCs. **A:** IHC with anti-CD34 (red) and anti-IgG or anti-Foxp1 (green) staining of hair follicles. **B:** IHC with anti-NFATc1 (green) and anti-Foxp1 (red) staining of hair follicle stem cells. Scale bars: 50 μm. Blue, DAPI.
(TIF)

S2 Fig IHC examination for the Foxp1 expression in hair follicles in the WT and *cKO* mice. IHC was performed with anti-Foxp1 in sections from the hair follicles of the *Foxp1* ^*fl/fl*^ (WT) and *K14-Cre; Foxp1* ^*fl/fl*^ (*cKO*) mice. The samples from the *cKO* were selected from anagen, catagen and telogen.
(TIF)

S3 Fig Ablation of Foxp1 shortens anagen duration during hair cycling. **A:** A dorsal view showed a sign of hair shedding in the *K14-Cre; Foxp1* ^*fl/fl*^ (*cKO*) mice at p29. **B:** Lengths of hair shafts plucked from the *cKO* mice at P47 were shorter than those of the WT (n = 4). *, p<0.05. **C:** HE staining for the first and second hair cycles in the WT and *cKO* mice at P45 to P55. **D:** A dorsal view for the first and second hair cycles in the WT and *cKO* mice from P22 to P55. **E:** The *cKO* mutant mice displayed shorter durations of telogen and anagen than WT controls following the first hair cycle.
(TIF)

S4 Fig TUNEL staining for the HaCat cells treated with one-hour stimulation of 500 μM H_2_O_2_. The TUNEL staining was performed in HaCat cells following one-hour stimulation with 500 μM H_2_O_2_.
(TIF)

S1 Table Primers of qRT-PCR. Primers were designed for qRT-PCR.
(DOCX)
