# Supplementary figures and images for: Foxp1 Regulates the Proliferation of Hair Follicle Stem Cells in Response to Oxidative Stress during Hair Cycling
Source: PLoS One. 2015 Jul 14;10(7):e0131674. doi: 10.1371/journal.pone.0131674 (PMC4501748; doi:10.1371/journal.pone.0131674)

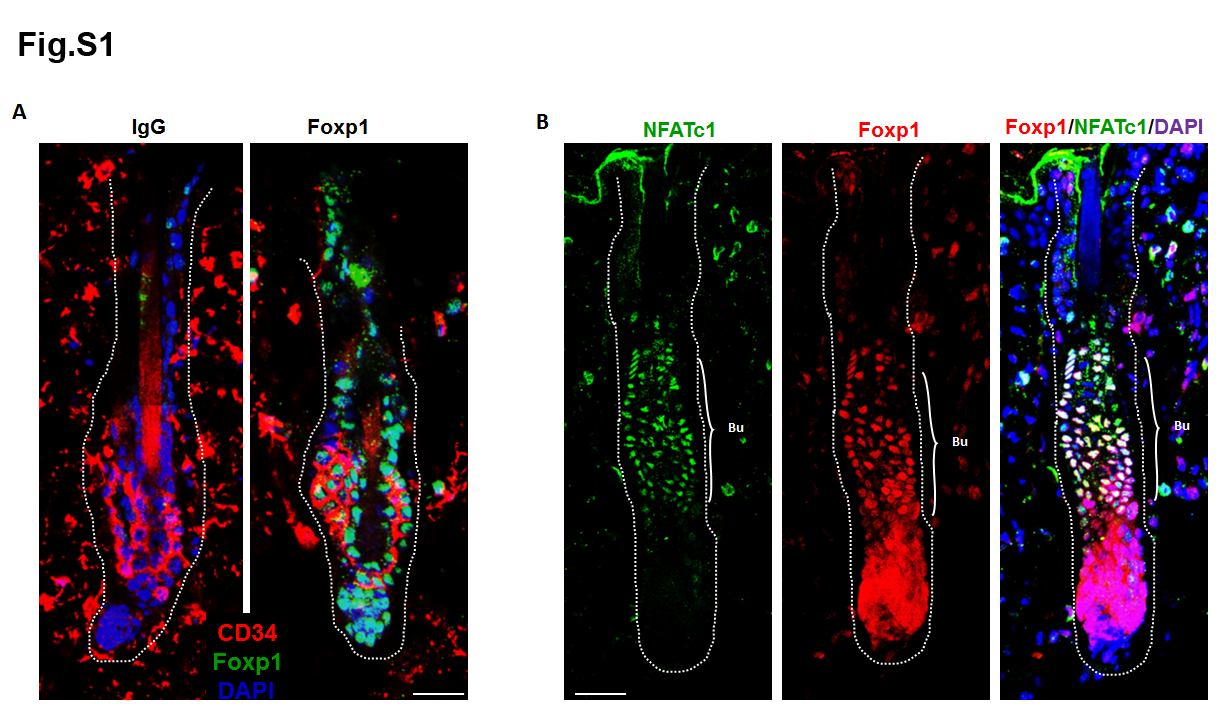

Supplement: S1 Fig — A: IHC with anti-CD34 (red) and anti-IgG or anti-Foxp1 (green) staining of hair follicles. B: IHC with anti-NFATc1 (green) and anti-Foxp1 (red) staining of hair follicle stem cells. Scale bars: 50 μm. Blue, DAPI. (TIF) [file pone.0131674.s002.tif]

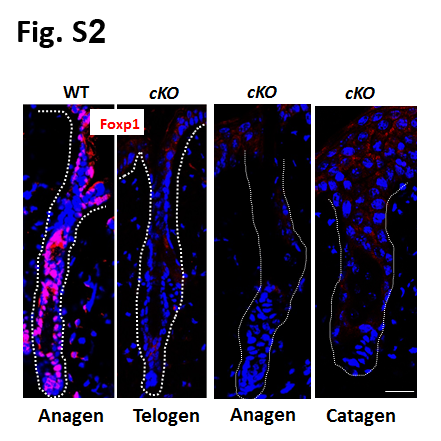

Supplement: S2 Fig — IHC was performed with anti-Foxp1 in sections from the hair follicles of the Foxp1 fl/fl (WT) and K14-Cre; Foxp1 fl/fl (cKO) mice. The samples from the cKO were selected from anagen, catagen and telogen. (TIF) [file pone.0131674.s003.tif]

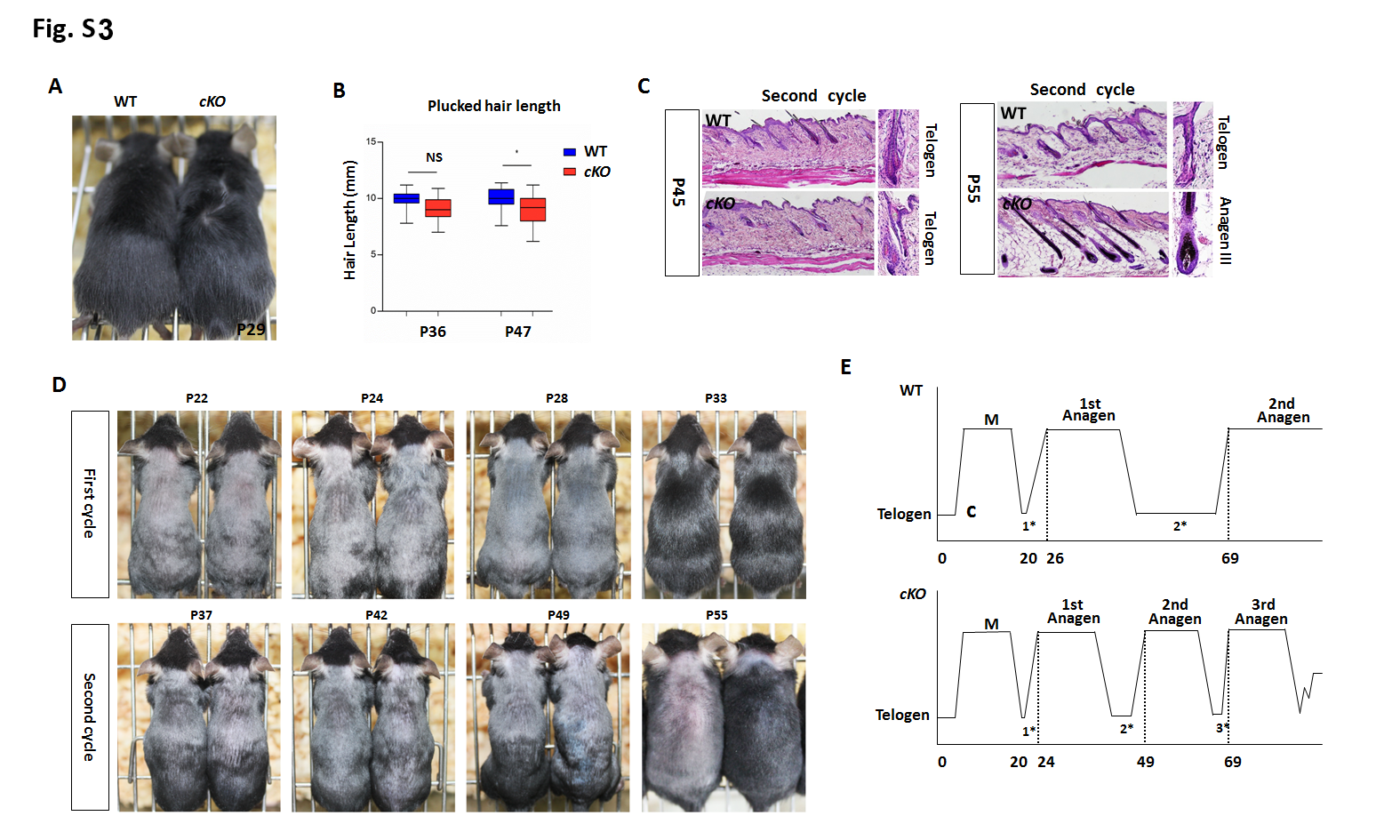

Supplement: S3 Fig — A: A dorsal view showed a sign of hair shedding in the K14-Cre; Foxp1 fl/fl (cKO) mice at p29. B: Lengths of hair shafts plucked from the cKO mice at P47 were shorter than those of the WT (n = 4). *, p<0.05. C: HE staining for the first and second hair cycles in the WT and cKO mice at P45 to P55. D: A dorsal view for the first and second hair cycles in the WT and cKO mice from P22 to P55. E: The cKO mutant mice displayed shorter durations of telogen and anagen than WT controls following the first hair cycle. (TIF) [file pone.0131674.s004.tif]

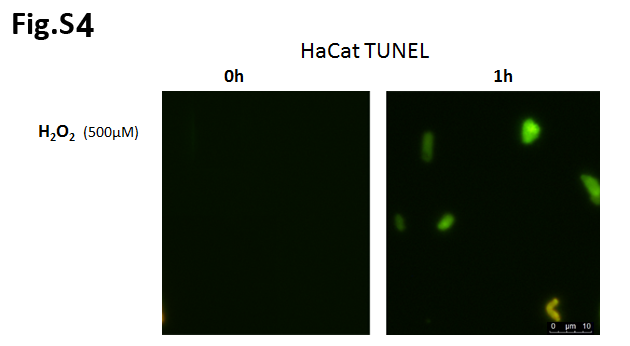

Supplement: S4 Fig — The TUNEL staining was performed in HaCat cells following one-hour stimulation with 500 μM H2O2. (TIF) [file pone.0131674.s005.tif]
