# Supplementary material for: Foxp1 Regulates the Proliferation of Hair Follicle Stem Cells in Response to Oxidative Stress during Hair Cycling
Source: PLoS One. 2015 Jul 14;10(7):e0131674. doi: 10.1371/journal.pone.0131674 (PMC4501748; doi:10.1371/journal.pone.0131674)
Supplement: S1 Table — Primers were designed for qRT-PCR. (DOCX) [file pone.0131674.s006.docx]

**Table. S1. Primers of qRT-PCR.**

Primers were designed for qRT-PCR.

| **Genes (mouse)** | **Forward primers** | **Reverse primers** |
| --- | --- | --- |
| GAPDH | 5’-AGAACATCATCCCTGCATCCA-3’ | 5’-CAGATCCACGACGGACACATT-3’ |
| Foxp1 | 5’- GTCTTGTGGCGTTCTGCA-3’ | 5’-GCTGGACCCGTTCTGGAT-3’ |
| p16 | 5’-GGGTTTCGCCCAACGCCCCGA-3’ | 5’-TGCAGCACCACCAGCGTGTCC-3’ |
| p19 ^ARF^ | 5’-ATCTGAGAAGCAGGGAACA-3’ | 5’- CCTTGAGTCCTTGTGGGT-3’ |
| p21 | 5’-TACTTCCTCTGCCCTGCTGC-3’ | 5’-GCTGGTCTGCCTCCGTTTT-3’ |
| p27 | 5’-ACTAACCCGGGACTTGGAGA-3’ | 5’- GAAATTCCACTTGCGCTGAC-3’ |
| p53 | 5’-TTCAGGCTTATGGAAACTAC-3’ | 5’-AGAAGGGACAAAAGATGACA-3’ |
| p66 | 5’-TTGAAAGGAAACGGAAAG-3’ | 5’- TCGGTATTGAACAGAGGC-3’ |
